# Development of Carvedilol-Loaded Albumin-Based Nanoparticles with Factorial Design to Optimize In Vitro and In Vivo Performance

**DOI:** 10.3390/pharmaceutics15051425

**Published:** 2023-05-06

**Authors:** Mohamed S. Attia, Mohamed F. Radwan, Tarek S. Ibrahim, Tarek M. Ibrahim

**Affiliations:** 1 Department of Pharmaceutics, Faculty of Pharmacy, Zagazig University, Zagazig 44519, Egypt; 2 Department of Pharmaceutical Chemistry, Faculty of Pharmacy, King Abdulaziz University, Jeddah 21589, Saudi Arabia

**Keywords:** carvedilol, healthcare, bovine serum albumin, nanoparticles, desolvation, factorial design, pharmacokinetic study

## Abstract

Carvedilol, an anti-hypertensive medication commonly prescribed by healthcare providers, falls under the BCS class II category due to its low-solubility and high-permeability characteristics, resulting in limited dissolution and low absorption when taken orally. Herein, carvedilol was entrapped into bovine serum albumin (BSA)-based nanoparticles using the desolvation method to obtain a controlled release profile. Carvedilol-BSA nanoparticles were prepared and optimized using 3^2^ factorial design. The nanoparticles were characterized for their particle size (Y_1_), entrapment efficiency (Y_2_), and time to release 50% of carvedilol (Y_3_). The optimized formulation was assessed for its in vitro and in vivo performance by solid-state, microscopical, and pharmacokinetic evaluations. The factorial design showed that an increment of BSA concentration demonstrated a significant positive effect on Y_1_ and Y_2_ responses with a negative effect on Y_3_ response. Meanwhile, the carvedilol percentage in BSA nanoparticles represented its obvious positive impact on both Y_1_ and Y_3_ responses, along with a negative impact on Y_2_ response. The optimized nanoformulation entailed BSA at a concentration of 0.5%, whereas the carvedilol percentage was 6%. The DSC thermograms indicated the amorphization of carvedilol inside the nanoparticles, which confirmed its entrapment into the BSA structure. The plasma concentrations of carvedilol released were observable from optimized nanoparticles up to 72 h subsequent to their injection into rats, revealing their longer in vivo circulation time compared to pure carvedilol suspension. This study offers new insight into the significance of BSA-based nanoparticles in sustaining the release of carvedilol and presents a potential value-added in the remediation of hypertension.

## 1. Introduction

As indicated by the World Health Organization (WHO), hypertension or elevated blood pressure is considered one of the primary causes of premature death worldwide, with a proportion of one hypertensive patient in four normal men and another one in five average women over a billion people [1]. Untreated hypertensive cases may predispose to persistent angina, heart attacks, and heart failure, which may cause unexpected death [2]. In addition to the lifestyle adjustment, medications approved by the US Food and Drug Administration (US-FDA) are often prescribed to control or reduce blood pressure, including angiotensin-converting enzyme inhibitors, calcium channel blockers, angiotensin II receptor blockers, diuretics, peripherally acting alpha-adrenergic blockers, beta-blockers, etc. [3].

A particular focus was devoted to the anti-hypertensive carvedilol, which has a non-cardioselective beta-blocker with a selective alpha-adrenergic blocking activity. Carvedilol acts by relaxing the blood vessels and slowing the heart rate, thereby reducing blood pressure and improving blood flow [4]. The oral administration of drugs is regarded as the most widely used and preferred route for patients because of its benefits, such as simplicity, convenience, painless administration, and self-application [5]. However, lipophilic drugs of low aqueous solubility can encounter poor pharmacokinetic profiles and low bioavailability while passing through the gastrointestinal tract (GIT). This observation aligns with the Biopharmaceutical Classification System (BCS), which categorizes carvedilol as a low-solubility and highly permeable drug. The highly lipophilic carvedilol faces a low oral bioavailability (25%) owing to its poor water solubility, in addition to its extensive first-pass metabolism [6]. Moreover, the pH variation along the GIT may restrain the pharmacological action of carvedilol owing to its pH-dependent solubility [2]. Therefore, many advanced oral drug delivery systems were investigated to enhance the aqueous solubility of carvedilol and reduce its adverse effects, for example, liposomes [7], self-microemulsions [8], cyclodextrin complexes [9], nanosuspensions [10], polymeric micelles [11], solid dispersions [12], etc.

A standard treatment plan for hypertension requires patients to take medications for several months or years to treat this ailment successfully. These patients may suffer from adverse effects following oral drug administration, such as poor drug solubility, pH variations, low intestinal resorption, and low bioavailability, in addition to those following the frequent drug dosage for a long time [13]. Hence, a potential drug delivery approach can tackle these problems by using a nano-drug delivery system to provide a sustained release of carvedilol when administered by a route other than the oral one. For instance, injectable sustained-release approaches have shown considerable clinical success in therapies for different diseases accompanied by reduced poor compliance or non-adherence to oral treatments [14]. Because blood pressure follows a circadian manner, sustained drug release techniques can be crucial in controlling blood pressure fluctuations. In addition, the entrapment of the drugs into the nanoparticulate systems can overcome the aforementioned concerns and help maintain the required drug concentration for sustained periods.

Several nanoparticle systems have been reported to be effective in treating the drawbacks of immediate-release anti-hypertensive drugs. In particular, polymeric nanoparticles can be made from polysaccharides [15], proteins [16], or synthetic polymers [17]. Proteins can produce good raw materials with good absorbability and low toxicity of their degradation end products [18]. The applicability of these protein-based nanoparticles is increasing due to their characteristics, including high storage stability, non-toxicity, non-antigenicity, and easy manufacture [16].

Albumin, the most abundant protein in the blood, is commonly used as a favorable component in the formulation of pharmaceutical preparations. Several attractive features of albumin make it an effective delivery vehicle for specific types of drugs with enhanced therapeutic effects [19]. It is characterized by its thermal stability, extensive stability over wide pH ranges, and ability to solubilize albumin-bound lipophilic drugs, thus enhancing their pharmacokinetic properties. There are two main sorts of albumin, human serum albumin (HSA) and bovine serum albumin (BSA). Both types participate in similar features with no observed variations corresponding to their high solubility in water, the long half-life, and similar molecular weight and amino acid residues [20]. The BSA has received wide acceptance owing to its multifaceted and unique features as a natural carrier and its biodegradability. The biocompatibility and non-immunogenicity of BSA can increase its stealthy property to escape from the reticuloendothelial system. Elzoghby et al. [18] reported that the injectable albumin-based nanoparticles could display no antigenicity or immunogenic responses, hence supporting their well-tolerance in vivo with no harmful adverse effects. Moreover, this type of albumin has several binding sites for hydrophilic and hydrophobic drugs and possesses the capability to decrease toxicity and increase the efficacy of drugs in addition to its ease of purification and low cost [21]. For this perspective, injectable BSA-based nanoparticles have been selected in our study to encapsulate and effectively deliver carvedilol and achieve an improved anti-hypertensive efficacy.

The BSA-based nanoparticles can be prepared by various techniques, such as desolvation, emulsification, thermal gelation, nano spray drying, self-assembly, and nanoparticulate albumin-bound technology [18,22]. The desolvation technique is a widespread procedure for developing BSA-based nanoparticles by which a desolvating agent is utilized to prepare well-defined nanoparticles with the denatured albumin and achieve the formation of spherical matrices. In addition, glutaraldehyde is commonly utilized as a crosslinker to ameliorate the stability of the prepared BSA-based nanoparticles [13]. This technique has gained a distinct superiority due to its desirable merits, including robustness, reproducibility, simplicity, encapsulation of hydrophilic and lipophilic drugs, and production of nanoparticles of smaller sizes and more controlled release than that of microparticles and liposomes, therefore improving the patient acceptance and compliance.

Studying the relationship between factors and results by the traditional one-factor-at-a-time (OFAT) strategy is time-consuming and tedious [23]. To obtain an optimal product, the quality by design (QbD) strategy is widely followed to focus on improving process efficiency and product development. This can be completed by identifying the impact of the process parameters and the formulation components on the critical quality attributes, therefore saving the time and the cost of the study [24]. Among several statistical techniques, the three-level factorial design was selected in our study to understand the relationship between the independent variables and the dependent responses, followed by the optimization process for preparing a reproducible product of desirable criteria.

Our study aimed to optimize a favorable parenteral BSA-based nanoparticle formulation loaded with carvedilol for achieving a prolonged release of carvedilol. The factorial design was embraced to investigate the impact of the main components on the critical quality attributes of BSA nanoparticles, such as particle size, entrapment efficiency, and in vitro drug release over days. The optimized formulation was injected intramuscularly into rats to estimate the pharmacokinetic evaluation of the drug in vivo. The long circulation effect of carvedilol-loaded BSA nanoparticles in vivo could ameliorate the bioavailability of carvedilol and provide better patient compliance due to the achieved lower frequency of drug dosing.

## 2. Materials and Methods

### 2.1. Materials

Carvedilol was kindly supplied from Global Napi Pharmaceuticals, 6th of October, Egypt. BSA was purchased from Sigma-Aldrich, St. Louis, MO, USA. Acetone was purchased from Scharlau, Barcelona, Spain. Triethyl amine and glutaraldehyde (25%) were purchased from Loba Chemie, Mumbai, India. Disodium hydrogen orthophosphate and potassium dihydrogen orthophosphate were purchased from El-Nasr Pharmaceutical Chemicals, Cairo, Egypt. Acetonitrile and methanol (HPLC grade) were purchased from Fisher Scientific, Loughborough, UK. The other chemicals were of analytical grade.

### 2.2. Methods

#### 2.2.1. Experimental Design and Optimization Process

In order to examine the relationship between the independent factors and the dependent responses and analyze the statistical data, the Design-Expert^®^ software (version 11, Stat-Ease Inc., Minneapolis, MN, USA) was utilized. A 2-factor, 3-level (3^2^) factorial design was set, and the target was to study the main and the interaction effects on the studied responses. The independent factors were BSA concentration (A) and carvedilol percentage in BSA nanoparticles (B). The dependent responses were particle size (Y_1_), entrapment efficiency (Y_2_), and time in days required to release 50% of carvedilol (T50) (Y_3_). Table 1 shows the three levels of the independent factors, and the goals required for the dependent responses are represented. In addition, Table 2 displays the nine experimental runs suggested by the design software under study. The polynomial equation shown below was generally followed to describe our models:

Y = b_0_ + b_1_A + b_2_B + b_3_AB + b_4_A^2^ + b_5_B^2^

where Y was the measured response, (b_0_) was the intercept of the polynomial equation, (b_1_–b_5_) were the regression coefficients of the dependent responses, (A and B) were the main terms, (AB) was the interaction term, and (A^2^ and B^2^) were the quadratic terms. This polynomial equation could be used to estimate the potential of the coefficients where the positive sign indicated the synergistic effect, while the negative sign indicated the antagonistic effect [25].

To conclude the significance of each term, the analysis of variance (ANOVA) and *p*-values with a 95% confidence interval (*p* < 0.05) were followed. Comparisons of different statistical parameters for evaluating the fitness of the data were made, including the multiple determination coefficient (R^2^), adjusted R^2^, predicted R^2^, and adequate precision. Normal plots of residuals, graphs of residual versus experimental runs, and graphs of predicted versus actual values were plotted to demonstrate the model’s adequacy. In addition, one-factor and three-dimensional (3D) response surface graphs were plotted to check the relationship and the interaction between the studied factors and the measured responses.

The optimization technique was utilized after the statistical analysis in order to optimize the formulation factors after preparing the carvedilol-loaded nanoparticles. Regarding the goal required for each dependent response (Table 1), the computed optimized BSA-based formulation with a desirability value ranging from zero to one. The high desirability value towards one could indicate the response compatibility to its desirable value [24]. Subsequently, the optimized formulation’s responses were studied once again, and the experimental values of the responses were compared with those predicted by the factorial design. Then, the prediction error percentage was calculated as follows [23]:

$$\mathrm{Prediction}\;\mathrm{error}\;\mathrm{percentage}\;=\frac{\mathrm{predicted}\;\mathrm{value}\;-\;\mathrm{experimental}\;\mathrm{value}\;}{\mathrm{predicted}\;\mathrm{value}}\;\times \;100$$

#### 2.2.2. Preparation of Carvedilol-Loaded BSA-Based Nanoparticles

The BSA-based nanoparticles loaded with carvedilol were prepared by a desolvation technique with procedures similar to those reported by Von Storp et al. [26] and Chen et al. [27] with some modifications. The BSA powder of different concentrations (0.5, 1, and 1.5%) was dissolved in 5 mL of double distilled water being previously adjusted to pH 8–9 using triethyl amine. The BSA solution was aggregated into nanoparticles through the dropwise addition of 20 mL of acetone containing different amounts of carvedilol under magnetic stirring (AREC F20500010, VELP Scientifica, Brianza, Italy). After the desolvation process, glutaraldehyde was added as a crosslinking agent at a ratio of 1 µL per 2 mg of BSA with continuous stirring for 2 h. Acetone was then evaporated using a rotary evaporator (Basis Hei-VAP, Heidolph Instruments GmbH, Schwabach, Germany) at −10 Pa, 35 °C, and 100 rpm. The BSA-based nanoparticles loaded with carvedilol were left dispersed into the original volume of water, and the dispersions were then subjected to ultrasonication for 2 min using a probe sonicator (GE 50, Scientific Engineering Inc., Woodbridge, VA, USA). The prepared formulations were lyophilized and stored at 4 °C for further evaluation.

#### 2.2.3. In Vitro Evaluation of Carvedilol-Loaded BSA-Based Nanoparticles

Particle size, polydispersity index (PDI), and zeta potential measurement. The particle size, PDI, and zeta potential of carvedilol-loaded albumin nanoparticles were measured using the dynamic light scattering (DLS) technique. A computerized Zetasizer (Nano–ZS90, Malvern Instruments Ltd., Malvern, UK) was applied to measure the nano-size particles. The samples of each formulation were suitably diluted with double distilled water at 25 °C prior to measurement. Moreover, the PDI values that reflected the size uniformity of the samples were monitored. The zeta potential values of each nanoparticle formulation were obtained by injecting the samples into a clear disposable zeta cell.

Entrapment efficiency measurement. The entrapment efficiency of carvedilol-loaded albumin nanoparticles was measured by separating the free carvedilol using the dialysis technique. The cellophane membranes were cut and hydrated in the receptor medium (phosphate buffer pH 7.4) for 2 h to ensure the complete wetting of the membranes. The formulations were redispersed in phosphate buffer pH 7.4, transferred into the dialysis bags (molecular weight cut-off 12,000–14,000 Da), and then put in glass bottles containing 100 mL of phosphate buffer pH 7.4 as a receptor medium. The bottles were shaken in a water bath shaker (SW-20C, Julabo Labortechnik GmbH, Seelbach, Germany) maintained at 25 °C and 100 rpm. At different time intervals, the receptor media were filtered using a 0.22 μm nylon syringe filter and the drug content was analyzed by spectrophotometer (Genesys 10S UV-VIS, Thermo Spectronic, Waltham, MA, USA) at λ_max_ 245 nm to determine the amounts of free drug. A fresh receptor medium was used after each measurement of drug content until no carvedilol was detected in the solution [28]. The entrapment efficiencies were calculated by the following equation as the ratio of the amount of the entrapped carvedilol to the total amount of the drug initially used.

$$\mathrm{Entrapment}\;\mathrm{efficiency}\;\mathrm{percentage}=\frac{\mathrm{Total}\;\mathrm{initial}\;\mathrm{drug}\;\mathrm{amount}-\mathrm{Free}\;\mathrm{drug}\;\mathrm{amount}\;}{\mathrm{Total}\;\mathrm{initial}\;\mathrm{drug}\;\mathrm{amount}}\times 100$$

In vitro cumulative drug release. The in vitro cumulative release of carvedilol from the prepared BSA-based nanoparticles was implemented using the cellophane membrane dialysis tubing method (molecular weight cut-off 12,000–14,000 Da). First, the cellophane membranes were cut and hydrated in the receptor medium (phosphate buffer pH 7.4) for 2 h. Second, the tested formulations were redispersed in phosphate buffer pH 7.4 and placed inside the dialysis bags sealed from the two ends. Third, the dialysis bags were transferred into closed bottles containing 100 mL of the aforementioned receptor medium. The bottles were carried to a thermostatically controlled water bath shaker maintained at 37 ± 1 °C and 100 rpm. Samples (3 mL) were withdrawn at different time intervals (0, 1, 2, 4 and 6 h) on the first day, and, subsequently, the process was repeated every day for a week. After sampling, an equal volume of fresh buffer was added to the receptor media to maintain the volume constant inside the bottles. The withdrawn sample was filtered using a 0.22 μm nylon syringe filter, and the drug content was assessed spectrophotometrically at λ_max_ 245 nm. The measurements were carried out three times and expressed as mean values ± standard deviation (SD).

Kinetic release study. The cumulative release data of carvedilol-loaded nanoparticles were examined for best fitting to different kinetic models. The zero-order, first-order, Higuchi, Hixson–Crowell, and Korsmeyer–Peppas models were studied using the following equations expressing each model, respectively (Q_t_ = K_o_.t, Q_t_ = 1 − e^−kt^, Q_t_ = K_H_.t^1/2^, Q_o_^1/3^ − Q_t_^1/3^ = K_HC_.t, and Q_t_/Q_∞_ = K_KP_.t^n^); where, Q_t_ was the drug amount released at the time (t); Q_o_ was the initial drug amount released; Q_∞_ was the drug amount released at time infinity (∞); K_o_, K, k_H_, K_HC_, and K_KP_ were the release rate constants of the previous models, respectively; and n was the release exponent. The model showing the highest R^2^ was considered the best model describing the release mechanism of carvedilol from the BSA-based nanoparticles. In addition, the n values of the Korsmeyer–Peppas model were used to confirm the release mechanism of the drug from the studied formulations [23]. The T50 values for the tested formulations were obtained according to the selected kinetic model.

#### 2.2.4. In Vitro Evaluation of Optimized Carvedilol-Loaded BSA-Based Nanoparticle Formulation

Transmission electron microscopy (TEM). The morphology of the optimized carvedilol-loaded BSA-based nanoparticle formulation was analyzed by using a transmission electron microscope. Gao et al. [29] described that a small drop of the optimized formulation was added to a carbon-coated grid after being diluted and dispersed in distilled water. It was kept for 2 min, and the excess liquid was dried using filter paper. A drop of 1% aqueous solution of phosphotungstic acid was added to the sample and then allowed to dry at room temperature. The sample was examined by TEM (JEM-2100, JEOL, Tokyo, Japan) at an accelerating voltage of 100 kV.

Differential scanning calorimetry (DSC). The thermal behaviors of pure carvedilol powder, BSA, carvedilol-free blank nanoparticle formulation, and optimized carvedilol-loaded BSA-based nanoparticle formulation were studied using a DSC instrument (DSC-60, Shimadzu, Japan). Samples were heated in sealed aluminum pans within the temperature range of 0–200 °C at a constant heating rate of 10 °C/min and under a nitrogen atmosphere with a 30 mL/min flow rate.

#### 2.2.5. In Vivo Evaluation of Optimized Carvedilol-Loaded BSA-Based Nanoparticle Formulation

Animals and ethical approval. Adult albino male rats of weights of 300~350 gm each were used. Before starting the experiments, the animals were kept in a 12 h light–12 h dark cycle at the ambient temperature for one week and received free water and food. The study was performed according to the Institutional Animal Care and Use Committee (IACUC) guidelines of the Faculty of Pharmacy, Zagazig University (Approval number: ZU-IACUC/3/F/184/2022; approval date: 29 August 2022).

High-performance liquid chromatography (HPLC) conditions. The HPLC analysis of plasma samples was performed for the separation of carvedilol. The mobile phase was a combination of 0.02 M monobasic potassium phosphate, acetonitrile, and methanol (32:28:40% *v*/*v*), and the pH was adjusted to 3.5 using orthophosphoric acid. The flow rate was set at 1 mL/min while the samples were injected into column C18 (4.6 × 250 mm, 5 μm), and the photodiode array detector was set at a wavelength was 245 nm.

Pharmacokinetic studies in rat plasma samples. The optimized BSA-based nanoparticle formulation was prepared. The rats were divided into two groups (*n* = 5) as follows:

Group I: Rats received a single intramuscular injection of pure carvedilol, a 20 mg/kg dose suspended in a phosphate buffer pH 7.4.

Group II: Rats received a single intramuscular injection of the optimized formulation equivalent to 20 mg/kg of carvedilol.

The blood samples were taken from the lateral tail veins of the rats into heparinized tubes at different time intervals (0.5, 1, 2, 4, 8, and 12 h) on the first day and then taken every day throughout the study period. The samples were centrifuged at 3000 rpm for 10 min to obtain plasma in which the drug concentrations were measured. Regarding the extraction of the drug from the rats’ plasma, each sample (0.5 mL) was added to 1.5 mL dichloromethane (DCM), vortexed, and centrifuged at 3000 rpm for 10 min. Then, 1 mL of DCM was withdrawn by a syringe and was left to evaporate at ambient temperature. The residuals were reconstituted with 0.5 mL methanol and then filtered by a syringe filter (0.22 µm) before injection into the Thermo Fisher Scientific^®^ HPLC system and Chromquest 5.0 software (Thermo Electron Corp., Bellefonte, PA, USA).

The pharmacokinetic parameters were calculated using the non-compartmental method by the Microsoft Excel add-in PKsolver program. The statistical approach of Student’s *t*-test was adopted and implemented via the GraphPad Prism^®^ program to analyze the measured pharmacokinetic parameters.

## 3. Results and Discussion

### 3.1. Preparation of Carvedilol-Loaded BSA-Based Nanoparticles

The GIT is recognized as the first physiological barrier to the absorption and delivery of most oral drugs. The oral bioavailability of the drugs is highly dependent on the solubility and stability of the drug in the GIT [30]. The action of carvedilol can be impeded mainly due to its high lipophilicity, poor solubility, and low oral bioavailability. Therefore, we developed injectable carvedilol-loaded BSA-based nanoparticles acting as biocompatible carriers to enhance the pharmaceutical action of carvedilol. The desolvation technique was followed using BSA owing to its outstanding benefits, including biocompatibility, biodegradability, non-immunogenicity, and safety. Throughout the desolvation process, the nanoparticles were generated by adding acetone to the BSA aqueous solution at high pH until a turbid solution was formed. According to the International Council on Harmonization Pharmaceuticals for Human Use (ICH) Q3C (R6) guideline, organic solvents are classified from class 1 to class 3 solvents. Acetone is a class 3 solvent and is approved by the ICH as a less toxic solvent with low risk to human health [31]. The BSA particles were not entirely stabilized and might reverse into the water phase. Thus, glutaraldehyde as a crosslinker was utilized to promote the stability of the formulated BSA-based nanoparticles by the incidence of the condensation reaction. The amine groups available in the side chains of BSA could interact with the aldehyde groups of glutaraldehyde [21]. Glutaraldehyde was observed to effectively form nanosized particles at this concentration since it crosslinks the 60 lysines amino acid of BSA at a saturation level of 138%. This was calculated following Langer et al. [32]. Moreover, these nanoparticles were prepared at high pH values because the particles might agglomerate, and no nanoparticles might be formed at low pH values during the desolvation time. Kufleitner et al. [33] reported that preparing the albumin nanoparticles at pH values above the isoelectric point of albumin (=4.9 of BSA) could increase the negative charges of the BSA molecules, making them less vulnerable to agglomeration, thus maintaining their stability. In addition, Aniesrani Delfiya et al. [34] pointed out that higher pH values could help reduce the size of the nanoparticles owing to the higher ionization of albumin existing above its isoelectric point, producing more repulsive nanoparticles.

### 3.2. Experimental Design and Statistical Analysis

Nine formulations were provided based on a 3^2^-factorial design, and their responses were characterized by varying the two independent factors within three levels. The measured responses for the prepared formulations were evaluated, and the impacts of main and interaction factors on the tested responses were studied.

#### 3.2.1. Effect of Independent Factors (A and B) on the Particle Size of Carvedilol-Loaded Nanoparticles (Y_1_ Response)

Concerning the statistical analysis of the particle size values (Table 3), the model was represented as a significant model due to its high F-value (164.01) and significant *p*-value (0.0007). In addition, the terms (A and B) have appeared as significant terms owing to their *p*-values (0.0004 and 0.0002, respectively). Meanwhile, other terms (AB, A^2^, and B^2^) did not establish significance. The R^2^ value could measure the variation amount around the mean described by the model. This model was found to be high (0.9964), which could indicate the model’s ability to detect about 99% of the variations [13]. The adequate precision could compare the range of the predicted values to the average prediction error. Its high value (above four) could reflect an adequate signal/noise ratio and express satisfactory model discrimination. The proper precision value was 39.87 in this model, indicating that the model could navigate the design space.

The polynomial equation developed by Y_1_ model was:

Y_1_ = 174.81 + 25.16 A + 33.48 B + 3.29 AB − 1.19 A^2^ + 6.04 B^2^

As reported by Hosny et al. [25], the coefficients of the factors could signalize the positive or negative influence of the factors on the measured responses. The polynomial equation of the Y_1_ model showed the positive effects of increasing the BSA concentration and the drug concentration on the particle size of the prepared nanoparticle formulations.

Furthermore, the normal plot of residuals was extracted from the design software, and the normal distribution of residuals (difference between actual and predicted values) on a straight line was evident to a substantial extent (Figure S1a Supplementary Material). Additionally, the externally studentized residuals versus runs were plotted where the points were present between the control limit lines (Figure S1b Supplementary Material). In addition, the actual values of the particle size and those predicted by the factorial design were plotted where there was a good correlation between these values, as shown in Figure S1c, Supplementary Material.

**Figure 1:**
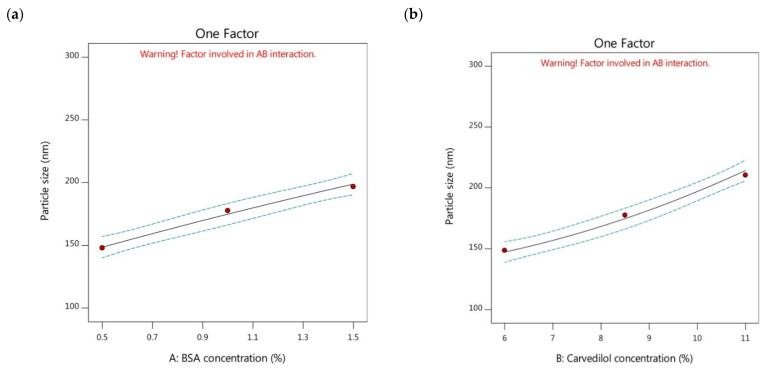
One factor plots of Y_1_ response. (**a**) effect of A on Y_1_; (**b**) effect of B on Y_1_.

By checking the model graphs, the particle size of the nanoparticles could be increased by increasing the BSA concentration (Figure 1a). By comparison of formulations possessing the same carvedilol percentage in BSA nanoparticles and different BSA concentrations, as shown in Table 2, it was found that the Y_1_ response could be minimized through the following order, F7 > F8 > F9, F4 > F3 > F2, and F6 > F5 > F1. The low particle size values were observed in the carvedilol-loaded nanoparticles of lower concentrations of BSA than those of higher concentrations of BSA. Perhaps, this could be explained by the fact that increasing the BSA concentration could increase the viscosity of the prepared BSA solution, and the transfer of protein between the water and the desolvation agent could decrease. Therefore, this could lead to slower nucleation rates and the formation of aggregated larger particles [35]. In addition, İnan and Özçimen [36] reported that high concentrations of BSA might cause coagulation of the BSA molecules, which could undergo electrostatic and hydrophobic interactions.

The particle size parameter of a drug delivery system could represent a fundamental key in drug transportation through systemic circulation. Nanoparticle systems with small particle sizes and hydrophilic surfaces could easily circulate in the blood over extended periods and sustain the duration of the pharmacological effect [13]. Rejinold et al. [37] found that an optimal nanoparticle size of 250 nm or lower, combined with highly positive or negative charges on the nanoparticle surface, can prevent rapid clearance by the reticuloendothelial system. Hence, it was noteworthy to observe that the particle size values of the studied nanoparticles ranged from 123.33 nm (F1) to 243.96 nm (F7), indicating the reproducibility of the BSA nanoparticles to affect the biodistribution of carvedilol positively [38].

Moreover, the PDI values could help measure the distribution homogeneity of the nanoparticle systems. As shown in Table S1, Supplementary Materia, lower PDI values of the nine carvedilol-loaded BSA-based nanoparticles were observed. This could elucidate the outstanding stability of the tested formulations and their feasibility of being injected with more uniform absorption in vivo [37]. Katona et al. [39] pointed out that nanoparticle systems of PDI values higher than 0.5 could not be recommended since they might result in an irregularity in the pharmacokinetic performance and variations in the therapeutic outputs.

On another side, the electrostatic interaction of the nanoparticles could be determined by studying the charge intensity on the particles’ surface. Zeta potential measurement is a useful tool for predicting the colloidal stability of nanoparticles during storage, as it can indicate the attractive and repulsive forces between the particles. The higher the zeta potential value, the lesser the aggregation of the nanoparticles and the higher the stability of the nanosystem [40]. According to the findings of Şenol et al. [41], a minimum value of 20 mV (either positive or negative) is recommended for nanoparticle systems to guarantee their electrostatic and steric stabilization. As per data reported in Table S1 (Supplementary Material), zeta potential values of the studied BSA nanoparticles ranged from −28.90 mV (F7) to −35.65 mV (F1). These findings could prove the convenient repulsion between the particles preventing their agglomeration, thus prolonging the circulation time and the nanoparticle’s stability. Moreover, the negative charges found on the surface of the system were related to the BSA end groups having negative charges above the isoelectric point of the BSA (=4.9). These negative charges could help preserve the stability of the BSA nanoparticles in vivo [37].

On the other hand, it could be observed the influence of changing the carvedilol percentage in BSA nanoparticles (factor B) on the particle size of the tested formulations. The increment of carvedilol percentage showed a significant increase in the size of the nanoparticles (Figure 1b). By comparison of formulations possessing the same BSA concentration and different drug concentrations as displayed in Table 2, it was found that the particle size decreased by the following sequence; F7 > F4 > F6, F8 > F3 > F5, and F9 > F2 > F1. The low particle size values were observed in the case of lower carvedilol percentage in BSA nanoparticles than in those systems with higher drug percentages. Gao et al. [29] reported that increasing the drug amount could increase the viscosity of the drug solution, making it difficult to disperse the components into the aqueous solution, leading to the formation of larger-sized nanoparticles.

The 3D response surface graphs were plotted to analyze the interaction between the studied factors and the measured responses by illustrating how changing the levels of two factors could significantly impact the response. Parallel lines could clarify the lack of interaction between the two factors. The interaction effects are represented in Figure S2 Supplementary Material.

#### 3.2.2. Effect of Independent Factors (A and B) on Entrapment Efficiency of Carvedilol-Loaded Nanoparticles (Y_2_ Response)

Concerning the ANOVA statistical analysis, the data presented in Table 3 mentioned that the high F-value (29.31) and low *p*-value (0.0095) of the Y_2_ model could indicate its significance. The A, B, and AB terms having *p*-values lower than 0.05 were considered significant. In contrast, the other terms of the model appeared as non-significant. In addition, the R^2^ was found to have a high value (0.9799). The precision value was higher than four (15.9429), reflecting the presence of an adequate signal/noise ratio and the capability of the model to navigate the design space. The polynomial equation developed by Y_2_ model was:

Y_2_ = 94.72 + 5.14 A − 4.25 B + 2.53 AB − 1.93 A^2^ − 1.60 B^2^

In this equation, increasing the BSA concentration could manifest its positive influence on the entrapment of carvedilol inside the tested nanoparticles, while the increment of carvedilol percentage showed an opposite trend. To confirm the fitness of the model and the minimal chance of error, the normal distribution of the residuals plot, the plot of externally studentized residuals versus runs, and actual versus predicted values are shown in Figure S3, Supplementary Material.

**Figure 2:**
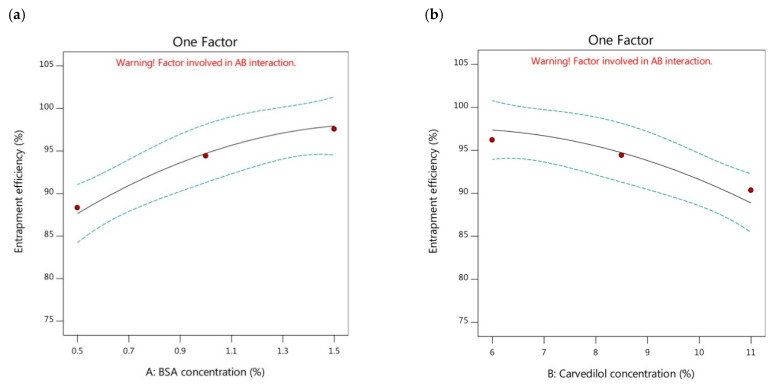
One factor plots of Y_2_ response. (**a**) effect of A on Y_2_; (**b**) effect of B on Y_2_.

The entrapment efficiency is recognized as a fundamental factor to indicate to what extent the prepared nanoparticles possess space for incorporating more amounts of drug and keeping them away from leakage. In turn, this can critically influence the drug release properties and its therapeutic effectiveness. This parameter can be defined as the ratio of the experimental drug content percentage to that of the theoretical outcome, and it particularly relies on the nature and the concentration of the utilized carrier [40]. As presented in Figure 2a, the entrapment efficiency of the BSA-based nanoparticles was significantly increased by the increment of the BSA concentration. By comparison of the formulations possessing the same carvedilol percentage and diverse BSA concentrations, as presented in Table 2, it was found that the reduction in the Y_2_ response could be demonstrated following this order, F6 > F5 > F1, F4 > F3 > F2, and F7 > F8 > F9. High amounts of carvedilol were observed to be encapsulated inside the BSA-based nanoparticles containing higher concentrations of BSA than those of lower BSA concentrations. This could be attributed to the increment of contact between the BSA molecules by increasing BSA concentration. Therefore, more BSA molecules could be available for encapsulating more carvedilol amounts in the binding sites of the BSA-based nanoparticles [42]. Moreover, the potential of increasing the BSA concentration in yielding a larger volume of the prepared nanoparticles could participate in holding more amounts of carvedilol; thus, greater entrapment efficiency could exist. These explanations were in alignment with the findings stated by Aniesrani Delfiya et al. [34].

Meanwhile, the impact of raising the carvedilol percentage in the BSA nanoparticles on the entrapment efficiency of the systems was investigated. The results showed that there was a significant decrease in the encapsulation efficiency of the nanoparticles with an increase in the percentage of carvedilol (Figure 2b). Further analysis of the data in Table 2 revealed that formulations with the same BSA concentration and different drug concentrations showed a decreasing trend in entrapment efficiency values as follows, F6 > F4 > F7, F5 > F3 > F8, and F1 > F2 > F9. The nanoparticles containing higher concentrations of carvedilol showed lower entrapment efficiencies than those with lower drug concentrations. This phenomenon could be attributed to the lack of available BSA molecules to create additional nanoparticles when higher amounts of carvedilol were added, resulting in an interaction between the excessive drug molecules and the existing BSA nanoparticles [43]. This interaction would lead to the formation of larger particles with lower entrapment efficiencies. This explanation could be consistent with the findings of Yang et al. [44], who reported that insufficient BSA molecules were unable to encapsulate the total amount of the drug when higher drug concentrations were used. Similar results were reported by Jose et al. [45].

On the other hand, the interaction effect (AB factor) could help clarify the influence of the interaction of two factors by altering their levels on the entrapment efficiency of the nanoparticles. As displayed by Figure S4 (Supplementary Materials), the term AB showed a significant effect on the entrapment efficiency parameter.

#### 3.2.3. Effect of Independent Factors (A and B) on T50 of Carvedilol-Loaded Nanoparticles (Y_3_ Response)

**Figure 3:**
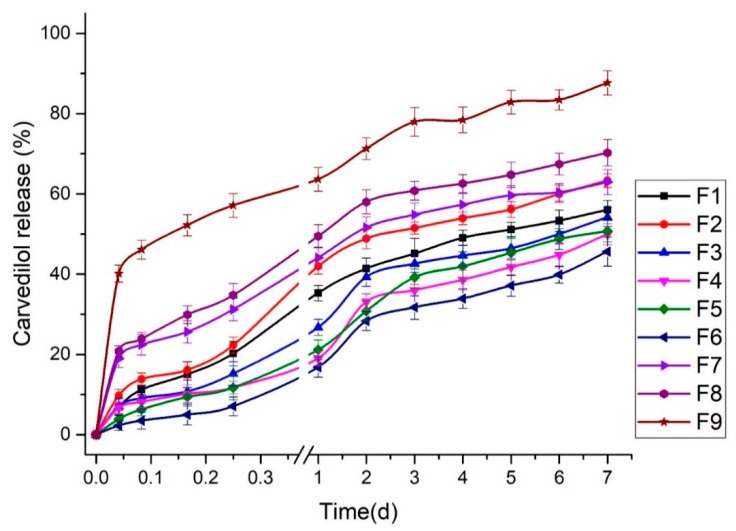
In vitro cumulative release of carvedilol from BSA-based nanoparticles (F1–F9).

The sustained release is fundamental for producing an excellent drug delivery system. BSA is considered one of the superior gatekeepers for preventing the fast release of drugs [45]. The in vitro cumulative release of carvedilol from the studied BSA-based nanoparticles (F1-F9) was assessed, as displayed in Figure 3. The profile of carvedilol release was observed as a biphasic release profile of two phases; the initial burst release of the drug in the first 6 h and the sustained drug release over days. Different initial burst release profiles were found among the studied formulations owing to the difference in the concentrations of the components utilized to prepare the nanoparticles. The burst release could be ascribed to the desorption and the diffusion of carvedilol from the outer surface of the BSA-based nanoparticles [13], where, the higher the amount of unentrapped carvedilol on the particle’s surface with no complete incorporation into the BSA, the faster the dissolution and the initial burst release of the drug [38]. Aside from this, continuous and sustained drug release could also be due to the slow carvedilol diffusion across the BSA matrix of the nanoparticle formulations by the solubilization of such carriers, including the degradation and erosion of the BSA. Gao et al. [29] reported that the incomplete paclitaxel release from the HSA-based nanoparticles might be related to the high affinity between the drug and the HSA in addition to the high entrapment of paclitaxel inside the nanoparticle formulations. In addition, the action of glutaraldehyde to crosslink with the BSA could reveal a slow release of the drug into the receptor medium making it an idealistic carrier for the sustained delivery of carvedilol [46].

Furthermore, the mechanism of carvedilol release from these formulations was determined by subjecting the in vitro release data to various mathematical kinetic models. The model that presented high R^2^ could be selected to best describe the drug release mechanism. As shown in Table 4, the carvedilol-loaded nanoparticle formulations showed higher R^2^ values corresponding to the Korsemeyer–Peppas model than other models. According to the n values for each formulation, the Korsemeyer–Peppas model could be employed to distinguish the following competing mechanisms; Fickian (diffusion-controlled) release mechanism when *n* ≤ 0.43, non-Fickian (anomalous) release mechanism when *n* = 0.43–0.85, and case II transport (relaxation-controlled) release mechanism when *n* ≥ 0.85 [47]. The data of Table 4 showed that F5 and F6 formulations demonstrated a non-Fickian/anomalous release mechanism indicating the incidence of both diffusion-controlled and swelling-controlled drug release from those nanoparticle formulations. However, the other formulations displayed n values less than 0.43, which could emphasize that the drug release fitted the Fickian diffusion mechanism (case I transport) [38].

The T50 values of the tested BSA nanoparticle formulations were also observed and then subjected to factorial analysis to study the effects of changing the studied factors on the T50 values of the formulations (Table 4). As per the data in Table 3, the high F-value (66.20) and the low *p*-value (0.0029) of the Y_3_ model could be observed, indicating its significance. The A, B, and B2 terms were significant (*p* < 0.05), while the AB and A2 terms were insignificant. The design software showed a high R^2^ value of 0.9910. Moreover, the adequate precision showed a high value (24.2133) greater than four, which resulted in a convenient signal/noise ratio and confirmed the model’s feasibility in expressing the design space. The polynomial equation developed by Y_3_ model was:

Y_3_ = 5.33 + 1.73 A − 2.57 B − 0.4850 AB − 0.0867 A^2^ − 1.44 B^2^

The equation shows that increasing the BSA concentration has positively impacted the T50 values, whereas increasing the percentage of carvedilol in the BSA nanoparticles resulted in the opposite effect. The model diagnostics are mentioned in the Supplementary Material (Figure S5).

**Figure 4:**
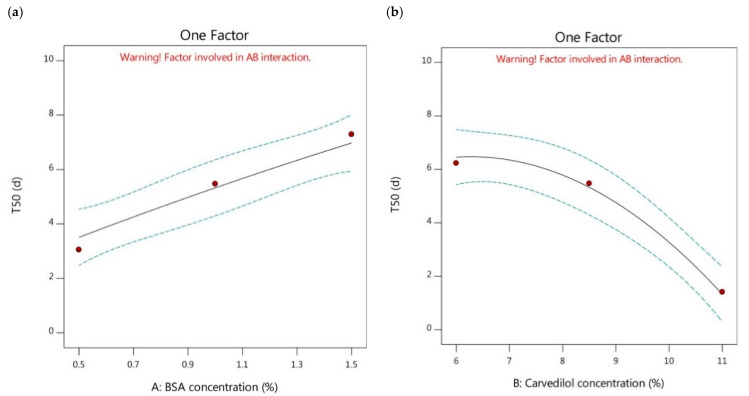
One factor plots of Y_3_ response. (**a**) effect of A on Y_3_; (**b**) effect of B on Y_3_.

Moreover, the effects of higher or lower concentrations of BSA on the cumulative release of carvedilol-loaded nanoparticles and, in turn, the T50 values were analyzed. It was revealed that increasing BSA concentration could significantly contribute to sustaining the carvedilol release profile by increasing the T50 values, as stated in Figure 4a. By comparison of the formulations having the same carvedilol percentage in the BSA nanoparticles and different BSA concentrations, as mentioned in Table 2, the T50 values could be reduced by the following order, F6 > F5 > F1, F4 > F3 > F2, and F7 > F8 > F9. The lower T50 values were observed in the carvedilol-loaded nanoparticles having lower BSA concentrations than those with higher BSA concentrations. This could be attributed to those particles of small size could have higher surface areas available for loading the drug into them and for the presence of the drug existing at the surface, thus resulting in faster release profiles with lower T50 values [40]. Therefore, controlling the particle size of the BSA-based formulations could be recognized as a crucial parameter that could directly influence the drug release rate. In addition, the increased entrapment efficiency induced by increasing the BSA concentration could result in forming more viscous solutions, which could assist in decreasing the release of the drug from the tested nanoparticles showing higher T50 values [34]. These explanations were in agreement with Abolhassani and Shojaosadati [43].

The effect of increasing the carvedilol percentage in the BSA nanoparticles on the release profile of the drug and the T50 values were also examined. The results showed that increasing carvedilol concentration significantly reduced the T50 values of the nanoparticles leading to less sustained release profiles (as depicted in Figure 4b). The comparison between the different formulations possessing the same BSA concentration and different carvedilol percentage in the BSA nanoparticles (Table 2) pointed out that the T50 values could be decreased as follows, F1 > F2 > F9, F5 > F3 > F8, and F6 > F4 > F7. The lower T50 values were observed in the case of higher carvedilol percentage in the BSA nanoparticles compared to those of lower drug concentration. This could be attributed to the reduced entrapment of carvedilol in the BSA-based nanoparticles exhibited after increasing the drug percentage with insufficient BSA molecules to create more nanoparticles. This might have led to the adhesion of excessive carvedilol molecules on the BSA surface, enabling burst release and lower T50 values [43].

On the other hand, the effect of the interaction factor (AB) on the T50 values of the drug-loaded nanoparticles was studied, as shown in Figure S6, Supplementary Material.

#### 3.2.4. Optimization Process

**Figure 5:**
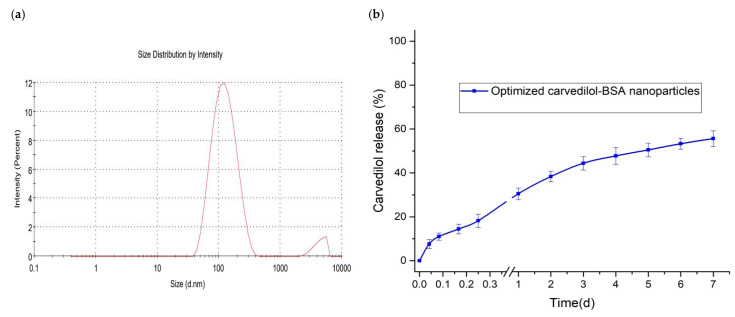
Characterization of optimized carvedilol-loaded BSA-based nanoparticle formulation. (**a**) particle size distribution; (**b**) in vitro cumulative drug release.

The optimization was carried out to reach favorable levels of the studied factors and achieve the target in preparing the BSA-based nanoparticles of the desired characteristics. According to the optimization targets listed in Table 1, an optimal nanoparticle formulation was suggested with a high desirability value of 0.847. This formulation consisted of BSA at a concentration of 0.5% and 6% carvedilol percentage in the BSA nanoparticles. The software predicted the values of the three responses as 124.32 nm for Y_1_ response, 92.82% for Y_2_ response, and 4.15 days for Y_3_ response. The optimized nanoparticle preparation was formulated and subjected to characterization, where the experimental results were found to be 123.54 nm, 91.61%, and 4.77 days for the three responses, respectively (Figure 5). The experimental and predicted values were in close agreement with acceptable prediction error percentages heralding the validity and the applicability of the experimental design models to assess the impact of the tested factors on the measured responses [23]. In addition, Table 4 shows that the mechanism of carvedilol release from the optimized formulation was best fitted to the Korsemeyer–Peppas model (R^2^ = 0.9959) following the Fickian (diffusion-controlled) release mechanism (*n* = 0.348).

### 3.3. In Vitro Evaluation of Optimized Carvedilol-Loaded Nanoparticle Formulation

#### 3.3.1. TEM Study

**Figure 6:**
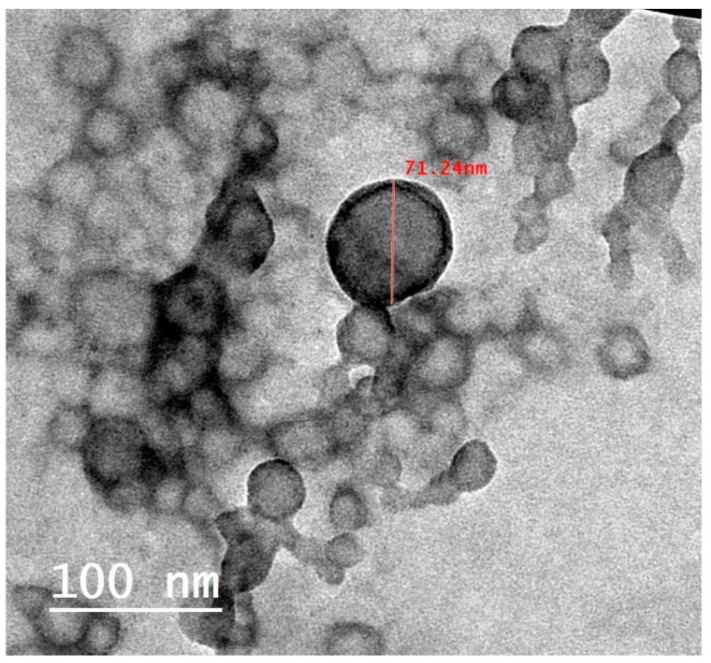
TEM image of optimized carvedilol-loaded BSA-based nanoparticle formulation.

The optimized formulation was visualized under a transmission electron microscope. As shown in Figure 6, the nanoparticles were observed to be spherical in their shapes with no aggregates. The results of the particle size values from the DLS technique were found to be relatively higher than those obtained by TEM analysis. This could be ascribed to the size measurement by TEM being performed on the tested nanoparticles at a dried state. On the contrary, the water molecules presented around the tested nanoparticles throughout the DLS measurement might overestimate the particle size values [19].

#### 3.3.2. DSC Study

**Figure 7:**
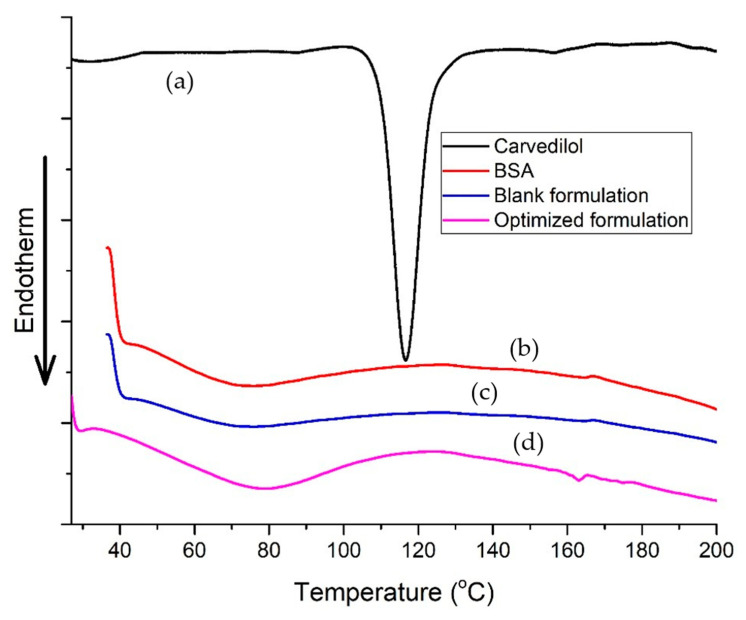
DSC thermograms of (**a**) pure carvedilol, (**b**) BSA, (**c**) blank optimized nanoparticle formulation, and (**d**) optimized carvedilol-loaded nanoparticle formulation.

The thermal behavior of the components could be studied by monitoring the presence, shifting, or disappearance of the endothermic or exothermic peaks. The alteration in the carvedilol crystallinity was observed, as shown in Figure 7. Pure carvedilol exhibited a distinct sharp endothermic peak at 113.03 °C, implying its crystallinity. The thermogram of BSA exhibited a broad enthalpy peak corresponding to heating the protein above its denaturation temperature, which was observed at 73.93 °C [19,48,49]. Upon analyzing the DSC profile of the optimized carvedilol-loaded BSA-based nanoparticles, we observed that the characteristic peak of carvedilol disappeared, suggesting that the drug incorporation in an amorphous state within the protein structure of the optimized formulation. Moreover, the endothermic peak corresponding to the BSA was shifted to 79.56 °C and ended at 117.35 °C. The detected shift in the peak for BSA denaturation in carvedilol-BSA formulation underscores the relevance of considering the various factors that impact the stability of protein formulations. According to previous reports on BSA aging by Farahnaky et al. [48], temperature exposure for two hours during nanoparticle preparation likely altered the protein structure. A shift in the peak may also have been caused by the stabilizing effects of carvedilol on BSA. This could imply the successful interaction and the entrapment of carvedilol inside the BSA nanoparticles, which might lead to a change in the molecular confirmation of the protein with the formation of a more organized and stable structure [49]. These results are supported by earlier studies in which various agents, such as limonene [50], halothane, and palmitic acid [51], increased the denaturation temperature of formed BSA complexes.

### 3.4. In Vivo Evaluation of Optimized Carvedilol-Loaded Nanoparticle Formulation

**Figure 8:**
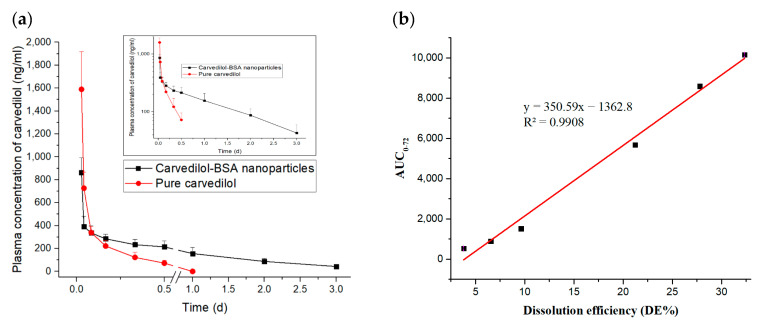
(**a**) Plasma concentration-time profiles of pure carvedilol and optimized carvedilol-loaded BSA-based nanoparticles following intramuscular administration to rats (*n* = 5). (**b**) In vitro/in vivo correlation (IVIVC) between DE% and AUC_0–72_.

The pharmacokinetic profiles of the pure carvedilol and the optimized carvedilol-loaded BSA-based nanoparticles after intramuscular injection into rats are shown in Figure 8a. Immediately following the pure carvedilol injection, the plasma drug concentration peaked at 1589.02 ng/mL within 30 min and then dramatically dropped, leaving less than 15% of the initial drug concentration within 4 h. This could suggest that carvedilol was quickly excreted by the rats. By contrast, the intramuscular administration of the optimized carvedilol-BSA nanoparticles showed a flattening of the pharmacokinetic profile compared to carvedilol alone. As a consequence of the above results, the amounts of the free carvedilol in the plasma were recorded in high concentrations in the group treated with pure carvedilol solution after 30 min; however, their amounts rapidly declined to an undetectable quantity following 12 h of administration. Contrary to this, it was still possible to determine the plasma concentrations of carvedilol released from the optimized BSA nanoparticles up to 72 h subsequent to their injection into rats. This could reveal the slower rate of clearance and the longer in vivo circulation time of the carvedilol-BSA nanoparticles than the pure drug suspension.

Moreover, it could be seen that the optimized carvedilol-BSA nanoparticles had a t_1/2_ value of 23.56 h, whereas the pure carvedilol was rapidly removed from the circulatory system with a short t_1/2_ of 4.34 h (Table 5). In light of the increased t_1/2_, the circulatory time of carvedilol after being encapsulated in the BSA-based nanoparticles was approximately increased by 5.43 times in comparison to that of carvedilol alone. The significant increase in the circulating time of the BSA nanoparticles in the blood was attributable to the robust attachment of carvedilol to BSA, as supported by the above-mentioned in vitro drug release and DSC investigations. Furthermore, the AUC_0–∞_ of the optimized carvedilol-BSA nanoparticles was increased by 3.22 folds than that of pure carvedilol (Table 5), which could lend credence to the effect of the BSA on extending the carvedilol release period in vivo [52].

Concerning the MRT values of both tested formulations, the optimized carvedilol-BSA preparation demonstrated a higher MRT value than that of the pure carvedilol suspension, indicating the long circulation action of carvedilol in vivo after being entrapped in the BSA-based nanoparticles, hence displaying a better bioavailability [47]. Additionally, the in vitro dissolution efficiency (DE%) of the optimized carvedilol-BSA nanoparticles was correlated to the in vivo AUC_0–72_ and the R^2^ value was 0.9908, as shown in Figure 8b. Our findings could signify the merits of using BSA nanoparticles in enhancing the carvedilol encapsulation in addition to sustaining the drug release in vitro and in vivo. This could reflect their possibility to be utilized for clinical purposes to improve the compliance of hypertensive patients.

## 4. Conclusions

The BSA-based nanoparticles had been investigated as promising strategies for carvedilol delivery owing to their intrinsic capability to deliver drugs of low water solubility at a sustained rate. The BSA in the nanoform displayed exemplary abilities to encapsulate carvedilol and achieve improved physicochemical and pharmacokinetic attributes. Per the data of the factorial design, the increment of the BSA concentration (Factor A) demonstrated its significant positive effect on the particle size (Y_1_), the entrapment efficiency (Y_2_), and the T50 values (Y_3_) of the studied nanoparticles, whereas factor B (carvedilol percentage in the BSA nanoparticles) represented its remarkable positive impact on the Y_1_ response in addition to its negative impact on the Y_2_ and Y_3_ responses. Choosing an optimized BSA-based nanoparticle formulation that could meet the required criteria proved the validity of the utilized design and the applicability of its findings. After injection into rats, the optimized carvedilol-loaded BSA-based nanoparticles showed their higher t_1/2_ and AUC values compared to those of the pure drug suspension, indicating their longer circulation time and better bioavailability in vivo. Our study could help acquire an adequately applicable and value-added nanoparticle product that would be beneficial in treating hypertensive patients.

## Figures and Tables

**Table 1:** Independent factors and dependent responses for carvedilol-loaded BSA-based nanoparticles.

Independent Factors	Unit	Symbol	Actual Levels (Coded)		
			Low (−1)	Medium (0)	High (+1)
BSA concentration	%	A	0.5	1	1.5
Carvedilol percentage in BSA nanoparticles	%	B	6	8.5	11
**Dependent responses**	**Unit**	**Symbol**	**Goal**		
Particle size	nm	Y_1_	Minimize		
Entrapment efficiency	%	Y_2_	Maximize		
T50	d	Y_3_	Target to 3.5 d		

BSA, bovine serum albumin; T50, the time required to release 50% of carvedilol.

**Table 2:** Nine experimental formulations as suggested by 3^2^ factorial design and observed values of responses.

Formulation	Actual Levels		Responses		
	A (%)	B (%)	Y_1_ (nm)	Y_2_ (%)	Y_3_ (d)
**F1**	0.5	6	123.33 ± 7.83	93.14 ± 0.97	4.46 ± 0.62
**F2**	0.5	8.5	147.84 ± 22.84	88.32 ± 1.03	3.05 ± 0.38
**F3**	1	8.5	177.56 ± 13.21	94.41 ± 0.77	5.47 ± 0.71
**F4**	1.5	8.5	196.66 ± 16.11	97.57 ± 0.91	7.29 ± 0.79
**F5**	1	6	148.60 ± 22.16	96.19 ± 1.31	6.23 ± 0.78
**F6**	1.5	6	167.83 ± 7.08	98.89 ± 1.02	8.51 ± 0.79
**F7**	1.5	11	243.96 ± 13.26	94.13 ± 0.98	2.25 ± 0.55
**F8**	1	11	210.36 ± 13.39	90.34 ± 0.42	1.41 ± 0.33
**F9**	0.5	11	186.30 ± 18.41	78.28 ± 1.25	0.14 ± 0.05

A, BSA concentration; B, carvedilol percentage in BSA nanoparticles; Y_1_, particle size; Y_2_, entrapment efficiency; Y_3_, the time required to release 50% of carvedilol (T50).

**Table 3:** ANOVA statistical results of responses (Y_1_, Y_2_, and Y_3_).

Source	Y_1_ Response			Y_2_ Response			Y_3_ Response		
	F-Value	*p*-Value	Significance	F-Value	*p*-Value	Significance	F-Value	*p*-Value	Significance
Model	164.01	0.0007	Significant	29.31	0.0095	Significant	66.20	0.0029	Significant
A	292.75	0.0004	Significant	76.26	0.0032	Significant	95.27	0.0023	Significant
B	518.14	0.0002	Significant	51.98	0.0055	Significant	208.89	0.0007	Significant
AB	3.34	0.1652	Non-significant	12.26	0.0394	Significant	4.97	0.1120	Non-significant
A^2^	0.217	0.6731	Non-significant	3.56	0.1555	Non-significant	0.0794	0.7964	Non-significant
B^2^	5.63	0.0983	Non-significant	2.48	0.2136	Non-significant	21.82	0.0185	Significant

ANOVA, analysis of variance; Y_1_, particle size; Y_2_, entrapment efficiency; Y_3_, time required to release 50% of carvedilol (T50); A, BSA concentration; B, carvedilol percentage in BSA nanoparticles.

**Table 4:** Kinetic release study of carvedilol-loaded BSA-based nanoparticle formulations.

Formulation	Zero Order Model	First Order Model	Higuchi Model	Hixson–Crowell Model	Korsmeyer–Peppas Model		
	R^2^	R^2^	R^2^	R^2^	R^2^	n	T50
F1	0.4897	0.6793	0.9095	0.6239	0.9854	0.327	4.46
F2	0.4309	0.6665	0.8847	0.5990	0.9789	0.313	3.05
F3	0.6357	0.7785	0.9559	0.7370	0.9880	0.374	5.47
F4	0.7322	0.8309	0.9781	0.8023	0.9910	0.412	7.29
F5	0.7837	0.8824	0.9909	0.8547	0.9951	0.447	6.23
F6	0.8429	0.9075	0.9882	0.8887	0.9882	0.503	8.51
F7	−0.0716	0.2968	0.6720	0.1896	0.9929	0.226	2.25
F8	−0.0883	0.3443	0.6621	0.2227	0.9915	0.224	1.41
F9	−1.1107	0.3320	0.0792	−0.2575	0.9962	0.140	0.14
Optimized	0.5762	0.7423	0.9436	0.6948	0.9959	0.348	4.77

R^2^, Coefficient of determination; n, release exponent; T50, the time required to release 50% of carvedilol.

**Table 5:** Pharmacokinetic parameters after intramuscular administration of pure carvedilol and optimized carvedilol-BSA nanoparticles to rats (*n* = 5). Data are presented as averages ± standard error of mean.

Parameters	Pure Carvedilol Suspension	Optimized Carvedilol-BSA Nanoparticles
t_1/2_ (h)	4.34 ± 0.77	23.56 ± 1.92
AUC_0–72_ (ng/mL.h)	3115.09 ± 784.53	10,154.75 ± 2652.49
AUC_0–∞_ (ng/mL.h)	3671.54 ± 1041.31	11,804.37 ± 3425.80
MRT (h)	4.98 ± 0.77	32.21 ± 3.03

BSA, bovine serum albumin; t_1/2_, elimination half life; AUC_0-72_, area under the curve from 0 to 72 h; AUC_0-∞_, area under the curve from 0 to infinity; MRT, mean residence time.

## Data Availability

Not applicable.

## Supplementary Materials

The following supporting information can be downloaded at: https://www.mdpi.com/article/10.3390/pharmaceutics15051425/s1, Figure S1: Model diagnostic plots of Y_1_ response including (a) normal plot of residuals (b) residuals versus runs plot (c) predicated versus actual values plot. Figure S2: 3D response surface graph showing the effect of AB factor on Y_1_ response. Figure S3: Model diagnostic plots of Y_2_ response including (a) normal plot of residuals (b) residuals versus runs plot (c) predicated versus actual values plot. Figure S4: 3D response surface graph showing the effect of AB factor on Y_2_ response. Figure S5: Model diagnostic plots of Y_3_ response including (a) normal plot of residuals (b) residuals versus runs plot (c) predicated versus actual values plot. Figure S6: 3D response surface graph showing the effect of AB factor on Y_3_ response. Table S1: Results of PDI and zeta potential values of carvedilol-loaded nanoparticles.

## References

[1] World Health Organization (WHO) (2021). Hypertension. https://www.who.int/news-room/fact-sheets/detail/hypertension..

[2] Alam T., Khan S., Gaba B., Haider M.F., Baboota S., Ali J. (2017). Nanocarriers as Treatment Modalities for Hypertension. Drug Deliv..

[3] Food and Drug Administration (FDA) (2022). Hypertension. https://www.fda.gov/consumers/minority-health-and-health-equity-resources/hypertension.

[4] Food and Drug Administration (FDA) (2021). High Blood Pressure–Understanding the Silent Killer. https://www.fda.gov/drugs/special-features/high-blood-pressure-understanding-silent-killer.

[5] Attia M.S., Hasan A.A., Ghazy F.S., Gomaa E. (2021). Solid Dispersion as a Technical Solution to Boost the Dissolution Rate and Bioavailability of Poorly Water-Soluble Drugs. Indian J. Pharm. Educ. Res..

[6] El-Say K.M., Hosny K.M. (2018). Optimization of Carvedilol Solid Lipid Nanoparticles: An Approach to Control the Release and Enhance the Oral Bioavailability on Rabbits. PLoS ONE.

[7] Nava G., Piñón E., Mendoza L., Mendoza N., Quintanar D., Ganem A. (2011). Formulation and In Vitro, Ex Vivo and In Vivo Evaluation of Elastic Liposomes for Transdermal Delivery of Ketorolac Tromethamine. Pharmaceutics.

[8] Ibrahim T.M., Abdallah M.H., El-Megrab N.A., El-Nahas H.M. (2018). Upgrading of Dissolution and Anti-Hypertensive Effect of Carvedilol via Two Combined Approaches: Self-Emulsification and Liquisolid Techniques. Drug Dev. Ind. Pharm..

[9] Cirri M., Mennini N., Maestrelli F., Mura P., Ghelardini C., Mannelli L.D. (2017). Development and In Vivo Evaluation of an Innovative “Hydrochlorothiazide-in Cyclodextrins-in Solid Lipid Nanoparticles” Formulation with Sustained Release and Enhanced Oral Bioavailability for Potential Hypertension Treatment in Pediatrics. Int. J. Pharm..

[10] Liu D., Xu H., Tian B., Yuan K., Pan H., Ma S., Yang X., Pan W. (2012). Fabrication of Carvedilol Nanosuspensions Through the Anti-Solvent Precipitation–Ultrasonication Method for the Improvement of Dissolution Rate and Oral Bioavailability. AAPS PharmSciTech.

[11] Öztürk K., Arslan F.B., Öztürk S.C., Çalış S. (2022). Mixed Micelles Formulation for Carvedilol Delivery: In-Vitro Characterization and In-Vivo Evaluation. Int. J. Pharm..

[12] Shamma R.N., Basha M. (2013). Soluplus^®^: A Novel Polymeric Solubilizer for Optimization of Carvedilol Solid Dispersions: Formulation Design and Effect of Method of Preparation. Powder Technol..

[13] Tazhbayev Y., Galiyeva A., Zhumagaliyeva T., Burkeyev M., Karimova B. (2021). Isoniazid—Loaded Albumin Nanoparticles: Taguchi Optimization Method. Polymers.

[14] Hobson J., Savage A., Dwyer A., Unsworth C., Arshad U., Pertinez H., Box H., Tatham L., Rajoli R.K., Neary M. (2021). Scalable Nanoprecipitation of Niclosamide and In Vivo Demonstration of Long-Acting Delivery After Intramuscular Injection. Nanoscale.

[15] Liu Z., Jiao Y., Wang Y., Zhou C., Zhang Z. (2008). Polysaccharides-Based Nanoparticles as Drug Delivery Systems. Adv. Drug Deliv. Rev..

[16] Tarhini M., Greige-Gerges H., Elaissari A. (2017). Protein-Based Nanoparticles: From Preparation to Encapsulation of Active Molecules. Int. J. Pharm..

[17] Tabasum S., Younas M., Zaeem M.A., Majeed I., Majeed M., Noreen A., Iqbal M.N., Zia K.M. (2019). A Review on Blending of Corn Starch with Natural and Synthetic Polymers, and Inorganic Nanoparticles with Mathematical Modeling. Int. J. Biol. Macromol..

[18] Elzoghby A.O., Samy W.M., Elgindy N.A. (2012). Albumin-Based Nanoparticles as Potential Controlled Release Drug Delivery Systems. J. Control. Release.

[19] Kandav G., Bhatt D.C., Jindal D.K., Singh S.K. (2020). Formulation, Optimization, and Evaluation of Allopurinol-Loaded Bovine Serum Albumin Nanoparticles for Targeting Kidney in Management of Hyperuricemic Nephrolithiasis. AAPS PharmSciTech.

[20] An F., Zhang X. (2017). Strategies for Preparing Albumin-Based Nanoparticles for Multifunctional Bioimaging and Drug Delivery. Theranostics.

[21] Karami E., Behdani M., Kazemi-Lomedasht F. (2020). Albumin Nanoparticles as Nanocarriers for Drug Delivery: Focusing on Antibody and Nanobody Delivery and Albumin-Based Drugs. J. Drug Deliv. Sci. Technol..

[22] Hassanin I., Elzoghby A. (2020). Albumin-Based Nanoparticles: A Promising Strategy to Overcome Cancer Drug Resistance. Cancer Drug Resist..

[23] Ibrahim T.M., Ayoub M.M., El-Bassossy H.M., El-Nahas H.M., Gomaa E. (2022). Investigation of Alogliptin-Loaded In Situ Gel Implants by 2^3^ Factorial Design with Glycemic Assessment in Rats. Pharmaceutics.

[24] Sarkar P., Bhattacharya S., Pal T.K. (2019). Application of Statistical Design to Evaluate Critical Process Parameters and Optimize Formulation Technique of Polymeric Nanoparticles. R. Soc. Open Sci..

[25] Hosny K.M., Rizg W.Y., Khallaf R.A. (2020). Preparation and Optimization of In Situ Gel Loaded with Rosuvastatin-Ellagic Acid Nanotransfersomes to Enhance the Anti-Proliferative Activity. Pharmaceutics.

[26] Von Storp B., Engel A., Boeker A., Ploeger M., Langer K. (2012). Albumin Nanoparticles with Predictable Size by Desolvation Procedure. J. Microencapsul..

[27] Chen Z., Hong G., Liu Z., Yang D., Kankala R.K., Wu W. (2020). Synergistic Antitumor Efficacy of Doxorubicin and Gambogic Acid-Encapsulated Albumin Nanocomposites. Colloids Surf. B Biointerfaces.

[28] Mokhtar M., Sammour O.A., Hammad M.A., Megrab N.A. (2008). Effect of Some Formulation Parameters on Flurbiprofen Encapsulation and Release Rates of Niosomes Prepared from Proniosomes. Int. J. Pharm..

[29] Gao Y., Nai J., Yang Z., Zhang J., Ma S., Zhao Y., Li H., Li J., Yang Y., Yang M. (2021). A Novel Preparative Method for Nanoparticle Albumin-Bound Paclitaxel with High Drug Loading and its Evaluation Both In Vitro and In Vivo. PLoS ONE.

[30] Fan N., Zhao J., Zhao W., Shen Y., Song Q., Shum H.C., Wang Y., Rong J. (2022). Biodegradable Celastrol-Loaded Albumin Nanoparticles Ameliorate Inflammation and Lipid Accumulation in Diet-Induced Obese Mice. Biomater. Sci..

[31] International Council on Harmonization (ICH) (2019). ICH Guideline Q3C (R6) on Impurities: Guideline for Residual Solvents. https://www.ema.europa.eu/en/documents/scientific-guideline/international-conference-harmonisation-technical-requirements-registration-pharmaceuticals-human-use_en-33.pdf.

[32] Langer K., Balthasar S., Vogel V., Dinauer N., Von Briesen H., Schubert D. (2003). Optimization of the Preparation Process for Human Serum Albumin (HSA) Nanoparticles. Int. J. Pharm..

[33] Kufleitner J., Worek F., Kreuter J. (2010). Incorporation of Obidoxime into Human Serum Albumin Nanoparticles: Optimisation of Preparation Parameters for the Development of a Stable Formulation. J. Microencapsul..

[34] Aniesrani Delfiya D.S., Thangavel K., Amirtham D. (2016). Preparation of Curcumin Loaded Egg Albumin Nanoparticles Using Acetone and Optimization of Desolvation Process. Protein J..

[35] Esim O., Gedik M.E., Dogan A.L., Gunaydin G., Hascicek C. (2021). Development of Carboplatin Loaded Bovine Serum Albumin Nanoparticles and Evaluation of its Effect on an Ovarian Cancer Cell Line. J. Drug Deliv. Sci. Technol..

[36] İnan B., Özçimen D. (2021). Microalgal Bioprocess Toward the Production of Microalgal Oil Loaded Bovine Serum Albumin Nanoparticles. Protein J..

[37] Rejinold N.S., Choi G., Piao H., Choy J. (2021). Bovine Serum Albumin-Coated Niclosamide-Zein Nanoparticles as Potential Injectable Medicine Against COVID-19. Materials.

[38] Wilson B., Selvam J., Mukundan G.K., Premakumari K.B., Jenita J.L. (2020). Albumin Nanoparticles Coated with Polysorbate 80 for the Targeted Delivery of Antiepileptic Drug Levetiracetam into the Brain. Drug Deliv. Transl. Res..

[39] Katona G., Balogh G.T., Dargó G., Gáspár R., Márki Á., Ducza E., Sztojkov-Ivanov A., Tömösi F., Kecskeméti G., Janáky T. (2020). Development of Meloxicam-Human Serum Albumin Nanoparticles for Nose-to-Brain Delivery via Application of a Quality by Design Approach. Pharmaceutics.

[40] Wen X., Huang X., Wu H. (2021). Development of a Novel Intraarticular Injection of Diclofenac for the Treatment of Arthritis: A Preclinical Study in the Rabbit Model. Acta Biochim. Pol..

[41] Şenol Y., Kaplan O., Varan C., Demirtürk N., Öncül S., Fidan B.B., Ercan A., Bilensoy E., Çelebier M. (2023). Pharmacometabolomic Assessment of Vitamin E Loaded Human Serum Albumin Nanoparticles on HepG2 Cancer Cell Lines. J. Drug Deliv. Sci. Technol..

[42] Kimura K., Yamasaki K., Nakamura H., Haratake M., Taguchi K., Otagiri M. (2018). Preparation and In Vitro Analysis of Human Serum Albumin Nanoparticles Loaded with Anthracycline Derivatives. Chem. Pharm. Bull..

[43] Abolhassani H., Shojaosadati S.A. (2019). A Comparative and Systematic Approach to Desolvation and Self-Assembly Methods for Synthesis of Piperine-Loaded Human Serum Albumin Nanoparticles. Colloids Surf. B Biointerfaces.

[44] Yang L., Cui F., Cun D., Tao A., Shi K., Lin W. (2007). Preparation, Characterization and Biodistribution of the Lactone form of 10-Hydroxycamptothecin (HCPT)-Loaded Bovine Serum Albumin (BSA) Nanoparticles. Int. J. Pharm..

[45] Jose P., Sundar K., Anjali C.H., Ravindran A. (2014). Metformin-Loaded BSA Nanoparticles in Cancer Therapy: A New Perspective for an Old Antidiabetic Drug. Cell Biochem. Biophys..

[46] Llabot J.M., Redin I.L., Agüeros M., Caballero M.J.D., Boiero C., Irache J.M., Allemandi D. (2019). In Vitro Characterization of New Stabilizing Albumin Nanoparticles as a Potential Topical Drug Delivery System in the Treatment of Corneal Neovascularization (CNV). J. Drug Deliv. Sci. Technol..

[47] Gomaa E., Eissa N.G., Ibrahim T.M., El-Bassossy H.M., El-Nahas H.M., Ayoub M.M. (2023). Development of Depot PLGA-Based In-Situ Implant of Linagliptin: Sustained Release and Glycemic Control. Saudi Pharm. J..

[48] Farahnaky A., Badii F., Farhat I.A., Mitchell J.R., Hill S.E. (2005). Enthalpy Relaxation of Bovine Serum Albumin and Implications for its Storage in the Glassy State. Biopolymers.

[49] Mohamed H.I., El-Kamel A.H., Hammad G.O., Heikal L.A. (2022). Design of Targeted Flurbiprofen Biomimetic Nanoparticles for Management of Arthritis: In Vitro and In Vivo Appraisal. Pharmaceutics.

[50] Chaturvedi S.K., Ahmad E., Khan J.M., Alam P., Ishtikhar M., Khan R.H. (2015). Elucidating the interaction of limonene with bovine serum albumin: A multi-technique approach. Mol. BioSyst..

[51] Nishimoto M., Matsuki H., Kaneshina S., Ogli K. (2005). Study on the Interaction between Bovine Serum Albumin and Inhalation Anesthetic Halothane by Differential Scanning Calorimetry.

[52] Yang Z., Du Y., Lei L., Xia X., Wang X., Tong F., Li Y., Gao H. (2023). Co-Delivery of Ibrutinib and Hydroxychloroquine by Albumin Nanoparticles for Enhanced Chemotherapy of Glioma. Int. J. Pharm..

