# Supplementary material for: Development of Carvedilol-Loaded Albumin-Based Nanoparticles with Factorial Design to Optimize In Vitro and In Vivo Performance
Source: Pharmaceutics. 2023 May 6;15(5):1425. doi: 10.3390/pharmaceutics15051425 (PMC10224401; doi:10.3390/pharmaceutics15051425)
Supplement: Supplementary file 1 [file pharmaceutics-15-01425-s001.zip › pharmaceutics-2287183-supplementary.pdf]

Supplementary Materials

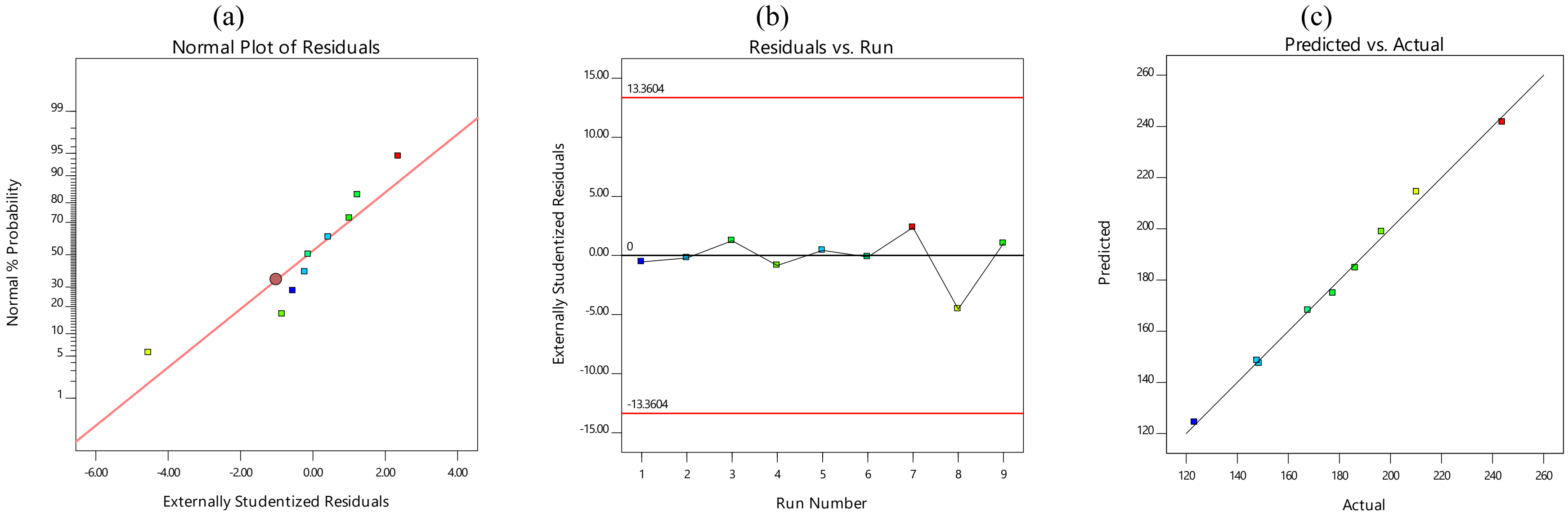

Figure S1: Model diagnostic plots of Y_1 response including (a) normal plot of residuals (b) residuals versus runs plot (c) predicated versus actual values plot.

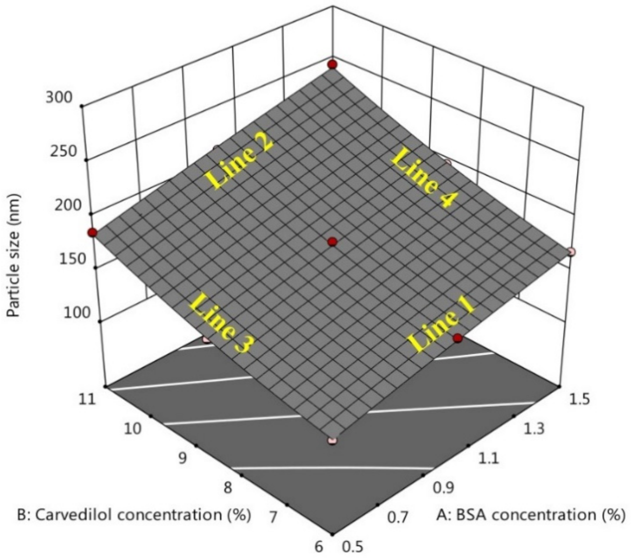

Figure S2: 3D response surface graph showing the effect of AB factor on Y_1 response.

Figure S2 represents that the particle size was increased by increasing the BSA concentration at the lowest drug concentration (6%) and at the highest carvedilol (11%), showing parallelism of lines 1 and 2. Likewise, the two parallel lines 3 and 4 showed that the rate of increasing the particle size by means of increasing the carvedilol percentage from 6% to 11% was not significant at the lowest or the highest concentration of BSA, respectively. Therefore, there was no interaction between the two studied factors, which could be compatible with the Table 3 results where the interaction term (AB) exhibited a non-significant action ($p = 0.1652$).

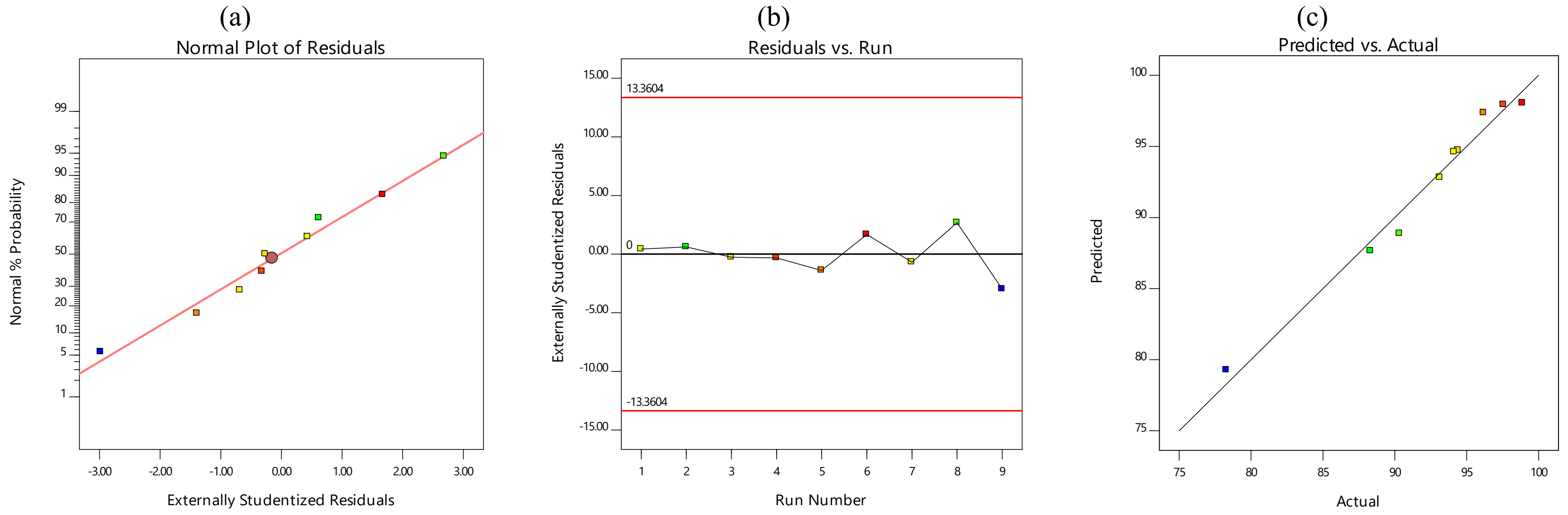

Figure S3: Model diagnostic plots of Y_2 response including (a) normal plot of residuals (b) residuals versus runs plot (c) predicated versus actual values plot.

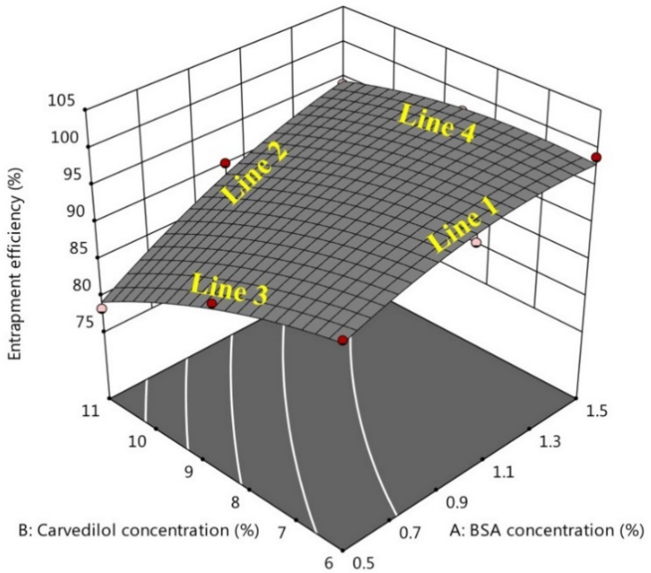

Figure S4: 3D response surface graph showing the effect of AB factor on Y_2 response.

As shown in Figure S4, it was observed that the increment rate of the entrapment of carvedilol in the BSA-based nanoparticles was significantly observed by increasing the BSA concentration and using the highest drug concentration (11%) when compared to the slighter increment in the drug entrapment induced by increasing the BSA concentration and using a lower drug concentration (6%). Moreover, the decrease in drug entrapment by increasing the drug percentage was remarkably monitored by using the lowest BSA concentration (0.5%) compared to the slight decrement in the drug entrapment exhibited by using the highest BSA concentration (1.5%). Thus, there was an obvious interaction effect between the A and B factors on the Y_2 response, as shown by the non-parallelism of each two opposite lines. This was in harmony with Table 3 results, where the term AB showed a significant effect with a p-value of 0.0394.

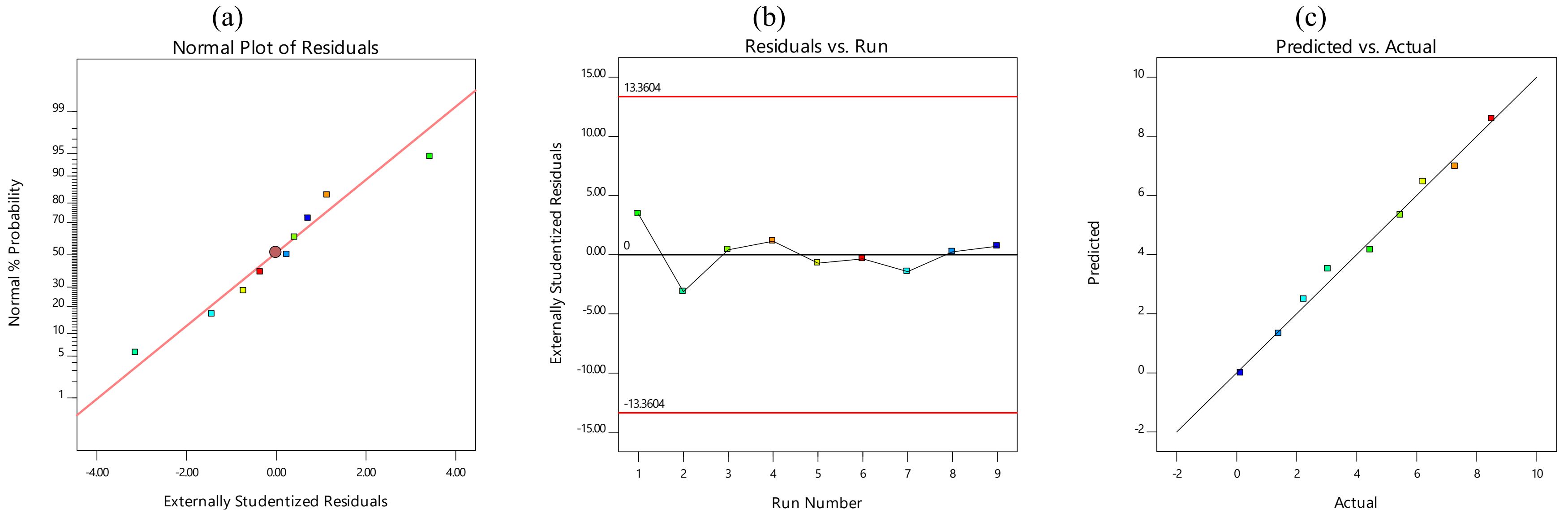

Figure S5: Model diagnostic plots of Y_3 response including (a) normal plot of residuals (b) residuals versus runs plot (c) predicated versus actual values plot.

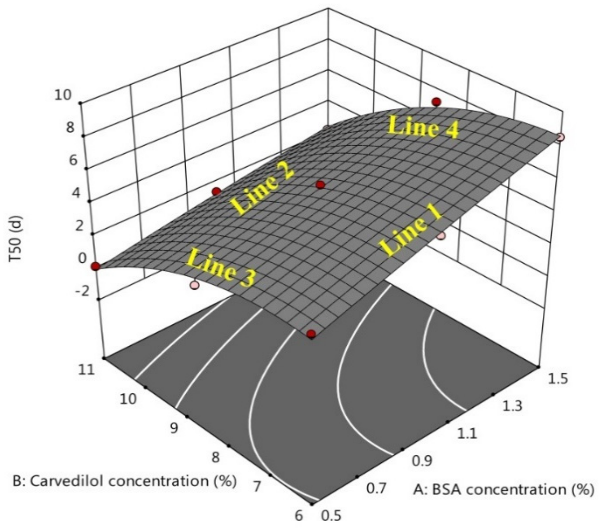

Figure S6: 3D response surface graph showing the effect of AB factor on Y_3 response.

There was no interaction effect between the two factors on the Y_3 response, where each two opposite lines were parallel to each other to a major extent. The absence of interaction could be supported by the Table 3 results, where the term AB showed an insignificant effect with a p-value of 0.2209.

Table S1: Results of PDI and zeta potential values of carvedilol-loaded nanoparticles.

Formulation	PDI	Zeta potential (mV)
**F1**	0.417 ± 0.13	-35.65 ± 1.21
**F2**	0.382 ± 0.09	-33.23 ± 1.10
**F3**	0.399 ± 0.08	-31.38 ± 0.39
**F4**	0.387 ± 0.15	-31.11 ± 0.91
**F5**	0.431 ± 0.08	-31.84 ± 1.01
**F6**	0.489 ± 0.11	-32.32 ± 1.50
**F7**	0.409 ± 0.09	-28.90 ± 0.78
**F8**	0.457 ± 0.10	-29.61 ± 0.97
**F9**	0.495 ± 0.12	-30.20 ± 0.56

PDI, poly dispersibity index; mV, millivolt.
